# S1PR3 inhibition protects against LPS-induced ARDS by inhibiting NF-κB and improving mitochondrial oxidative phosphorylation

**DOI:** 10.1186/s12967-024-05220-9

**Published:** 2024-06-05

**Authors:** Junnan Peng, Rui Tang, Jing He, Qian Yu, Daoxin Wang, Di Qi

**Affiliations:** Department of Respiratory and Critical Care Medicine, Second Affiliated Hospital of Chongqing Medical University, No.76 Linjiang Road, Yuzhong District, Chongqing, 400010 People’s Republic of China

**Keywords:** S1PR3, ARDS, NF-κB, Mitochondrial oxidative phosphorylation, Endothelial cell

## Abstract

**Background:**

Inflammation and endothelial barrier dysfunction are the major pathophysiological changes in acute respiratory distress syndrome (ARDS). Sphingosine-1-phosphate receptor 3 (S1PR3), a G protein-coupled receptor, has been found to mediate inflammation and endothelial cell (EC) integrity. However, the function of S1PR3 in ARDS has not been fully elucidated.

**Methods:**

We used a murine lipopolysaccharide (LPS)-induced ARDS model and an LPS- stimulated ECs model to investigate the role of S1PR3 in anti-inflammatory effects and endothelial barrier protection during ARDS.

**Results:**

We found that S1PR3 expression was increased in the lung tissues of mice with LPS-induced ARDS. TY-52156, a selective S1PR3 inhibitor, effectively attenuated LPS-induced inflammation by suppressing the expression of proinflammatory cytokines and restored the endothelial barrier by repairing adherens junctions and reducing vascular leakage. S1PR3 inhibition was achieved by an adeno-associated virus in vivo and a small interfering RNA in vitro. Both the in vivo and in vitro studies demonstrated that pharmacological or genetic inhibition of S1PR3 protected against ARDS by inhibiting the NF-κB pathway and improving mitochondrial oxidative phosphorylation.

**Conclusions:**

S1PR3 inhibition protects against LPS-induced ARDS via suppression of pulmonary inflammation and promotion of the endothelial barrier by inhibiting NF-κB and improving mitochondrial oxidative phosphorylation, indicating that S1PR3 is a potential therapeutic target for ARDS.

**Supplementary Information:**

The online version contains supplementary material available at 10.1186/s12967-024-05220-9.

## Background

Acute respiratory distress syndrome (ARDS) is a life-threatening illness characterized by diffuse inflammation and pulmonary oedema [1]. ARDS is the leading cause of death in critically ill patients, and the incidence of ARDS sharply increased during the coronavirus disease 2019 pandemic [2]. The prognosis of patients with ARDS has improved in recent years due to increased awareness and effective supportive therapy [1, 2]. However, ARDS-related mortality remains unacceptably high, as shown by a recent study in which the 60-day mortality was up to 44.1% among those with severe ARDS [3]. Currently, effective treatments for ARDS are still lacking, and ARDS remains one of the major public health burdens worldwide.

Although the pathophysiology of ARDS is complex, an excessive inflammatory response and endothelial barrier dysfunction have been proven to be the two major pathophysiological changes in ARDS [4]. Endothelial cells (ECs) line the luminal surface of the vasculature, forming a semipermeable barrier that separates the blood from the underlying tissue [5]. In ARDS, endothelial barrier injury due to various stimuli leads to increased lung vascular permeability, resulting in alveolar flooding and interstitial oedema, and contributing to the high mortality of ARDS patients [6]. Inflammation is also an important mechanism of pulmonary ARDS pathogenesis. In ARDS, a dysregulated inflammatory response activates various inflammatory cells in the lung, releasing large quantities of inflammatory mediators and cytokines. These factors increase alveolar-capillary permeability, leading to pulmonary oedema and hypoxemia, which are critical hallmarks of ARDS [7]. Thus, an effective treatment strategy for ARDS would be to suppress inflammation and restore the endothelial barrier.

Sphingosine 1-phosphate (S1P) is a bioactive lipid that regulates multiple biological responses [8]. S1P exerts its effect by binding to specific G-protein coupled receptors (S1PR1-5). Multiple G proteins and downstream effectors bind to these receptors and trigger various cell responses [9]. Among these five S1PRs, S1PR3 is involved in the regulation of inflammation and vascular barrier function in some diseases, such as sepsis [10, 11] and brain ischaemia–reperfusion injury [12]. To date, several studies have explored the association between S1PR3 and ARDS [13–16]. Viriyavejakul et al. [14] reported that S1PR3 is overexpressed in the lung tissues of severe plasmodium falciparum malaria patients with ARDS. Sun et al. [15] reported that plasma S1PR3 concentrations are significantly increased in patients with sepsis-induced ARDS and higher levels are associated with increased mortality. Furthermore, they found that two single nucleotide polymorphisms of the S1PR3 have a significant association with a decreased risk for sepsis-associated ARDS [16]. Taken together, these clinical studies indicated that S1PR3 may be a promising candidate for the treatment of ARDS. However, the effects of S1PR3 inhibition on inflammation and the endothelial barrier in ARDS and its potential mechanism are still unclear.

In this study, we first examined the S1PR3 expression level in lung tissues of lipopolysaccharide (LPS)-induced ARDS mice. Then, we investigated the effect of S1PR3 inhibition on inflammation and endothelial barrier function in the LPS-induced ARDS mouse model and the LPS-stimulated ECs model. Finally, we explored the possible mechanisms by which S1PR3 inhibits ARDS and validated them in vivo and in vitro.

## Materials and methods

### Mice

Male C57BL/6 mice (8 to 10 weeks old, 22–24 g) were obtained from the Experimental Animal Center of Chongqing Medical University. The mice had ad libitum access to standard rodent chow and autoclaved drinking water in a dedicated housing room with a 12/12 h light/dark cycle (8 am-8 pm). The Ethics Committee of the Second Affiliated Hospital of Chongqing Medical University approved all animal experiments following the National Institutes of Health Guide for the Care and Use of Laboratory Animals. The animal operators were not blinded while the investigators responsible for data collection and analysis were blinded during the animal experiments.

### Reagents

Lipopolysaccharide (LPS, *Escherichia coli* serotype 055: B5) and Hoechst 33258 were obtained from Sigma-Aldrich (St. Louis, MO, USA). TY-52156, FITC-conjugated dextran, MitoSOX, JC-1, NF-κΒ activator 2 and oligomycin were obtained from MedChemExpress (MCE; Monmouth Junction, NJ, USA). A terminal deoxynucleotide transferase-mediated dUTP nick end labelling (TUNEL) apoptosis assay kit was obtained from Roche (Basel, Switzerland). Mouse/human interleukin (IL)-6 and tumor necrosis factor (TNF)-α enzyme-linked immunosorbent assay (ELISA) kits were obtained from Ruixin Biotechnology (Fujian, China). The total protein extraction kit was obtained from Bestbio (Shanghai, China). The 4 × SDS‒PAGE loading buffer was obtained from TaKaRa (Kusatsu, Japan). Anti-S1PR3, anti-VE-cadherin, anti-β-catenin, anti-VCAM-1, anti-p-IKBα and anti-IKBα antibodies were obtained from Abcam (Cambridge, UK). Anti-LY6G, anti-p-IKKβ, anti-IKKβ, anti-p-NF-kB, anti-NF-kB, anti-GAPDH, HRP-conjugated secondary antibodies and rhodamine-conjugated phalloidin were obtained from ABclonal (Wuhan, China). RPMI 1640 medium and foetal bovine serum were obtained from Gibco (USA). An enhanced chemiluminescence (ECL) kit, Lipofectamine 3000 reagent, Alexa Fluor 488-conjugated goat anti-rabbit IgG antibody and TRIzol reagent were obtained from Invitrogen (CA, USA). Evans blue dye, crystal violet solution, an H&E staining kit, an adenosine triphosphatase (ATP) assay kit and the mitochondrial respiratory complex activity assay kits were obtained from Solarbio (Beijing, China). A bicinchoninic acid (BCA) protein quantification kit and penicillin–streptomycin, dihydroethidium (DHE) and 4',6-diamidino-2-phenylindole (DAPI) solutions were obtained from Beyotime (Shanghai, China). An Annexin V-FITC / PI apoptosis detection kit was obtained from Elabscience (Wuhan, China). The S1PR3-shRNA virus (pAAV-U6-shRNA (S1PR3)-CMV-EGFP-WPRE) and controls were constructed by OBIO Technology (Shanghai, China) (primer sequences available upon request). S1PR3-targeting small interfering RNA (siRNA) and control siRNA were purchased from Genepharma incorporation (Shanghai, China) (primer sequences available upon request).

### LPS-induced acute lung injury model

In this experiment, a total of 114 mice were used. Mice were injected intraperitoneally with 2% pentobarbital in PBS (50 mg/kg), and then 1 mg/mL LPS (5 mg/kg body weight) or phosphate-buffered saline (PBS) was injected into the trachea via a 20-gauge catheter. We attached the catheter to a mouse ventilator and then assessed chest expansion to confirm the correct catheter placement. The mice were kept in a vertical position for 2 min after intratracheal instillation to ensure the distribution of the LPS or PBS in the lungs. TY-52156 is a selective S1PR3 inhibitor, and TY-52156 (10 mg/kg) was injected intraperitoneally 1 h before LPS injection. For the adenovirus-associated virus (AAV) injection experiments, NF-κΒ activator 2 (1 mg/kg) or oligomycin (0.5 mg/kg) was injected intraperitoneally 30 min before LPS/PBS injection. We collected lung tissue, bronchoalveolar lavage fluid (BALF) and serum at 48 h after LPS administration using methods described previously [17]. All the samples were stored at − 80 °C until analysis.

### BALF

Mice were anaesthetized, and a 20-gauge catheter was inserted into the trachea. Then, a syringe was connected to the catheter, and the lungs were lavaged three times with 1 mL of cold sterile PBS to collect BALF [18]. Then, the BALF was centrifuged (800 rpm, 15 min, 4 ℃), the cells were counted, and the supernatant was stored for the analysis of protein concentrations and inflammatory factors. A BCA protein assay kit was used to determine the protein concentrations in the BALF supernatants. The precipitate was resuspended, and the total number of cells in the BALF was determined with a cell counting instrument. The number of neutrophils in the BALF was examined using Wright‒Giemsa staining [19].

### Hematoxylin and eosin (H&E) staining and lung injury score

According to the protocol of the H&E staining kit, lung tissues were paraffin-embedded, dewaxed, rehydrated, and stained with H&E. The stained sections were mounted on slides and evaluated with a light microscope (BX51 Olympus, Tokyo, Japan). The severity of lung injury was evaluated using a semiquantitative scoring methodology as previously described [20, 21]. Specifically, the severity of lung injury includes four independent variables: oedema, haemorrhage, infiltration or aggregation of neutrophils in the alveolar cavity or vascular wall, thickening of the alveolar wall and/or formation of a hyaline membrane. The grading scale to score the pathological findings was as follows: 0 = no injury; 1 = modest and limited injury (< 25%); 2 = intermediate injury (25–50%); 3 = widespread or prominent injury (50–75%); and 4 = widespread and most prominent injury (> 75%). The results were graded from 0 to 4 for each item. The four variables were summed to represent the lung injury score (total score: 0–16). The lung injury score was calculated by 2 different researchers. We took the average scores if the 2 researchers arrived at different values.

### Lung wet/dry ratio

We weighed the right upper lung tissues immediately after removal (wet weight) and then dried them in an oven at 60 °C for 48 h (dry weight). The wet weight was divided by the dry weight to calculate the wet weight/dry weight ratio.

### Vascular permeability measurements in the lung

Mice were intravenously injected with Evans blue dye (EBD; 30 mg/kg), euthanized after 30 min, and perfused with PBS for 2 min. The extracted lung tissues were weighed, homogenized in 1 mL of PBS, and extracted in 2 mL of formamide overnight at 60 °C. The tissue EBD concentration (μg EBD/g wet lung tissue) was determined by measuring absorbance at 620 nm.

### Immunohistochemistry (IHC)

IHC was performed on paraffin-embedded lung tissue as previously described [22]. LY6G antibody was used at a dilution of 1:200 at 4 °C overnight.

### Lung tissue immunofluorescence

Immunofluorescence staining of lung tissue sections was performed as previously described [23] on paraffin-embedded lung tissue. The sections were incubated with anti-VE-cadherin (1:500 dilution) or anti-β-catenin (1:250 dilution) antibodies at 4 °C overnight. An anti-rabbit Alexa Fluor 488-conjugated secondary antibody (1:1000 dilution) was used for immunofluorescence detection.

### TUNEL assay

Paraffin-embedded lung tissue sections were subjected to a TUNEL assay. An in situ cell death detection kit was used to detect apoptotic cells following the manufacturer's instructions. TUNEL-positive cells were counted under a fluorescence microscope (BX53 Olympus, Tokyo, Japan).

### mRNA sequencing

Transcriptome sequencing and analysis were conducted by OE Biotech Co., Ltd. (Shanghai, China). Total RNA was extracted from lung tissues using TRIzol reagent, the library was constructed using the VAHTS Universal V6 RNA-seq Library Prep Kit, and sequencing was performed using the Illumina NovaSeq 6000 platform (Illumina, CA, USA). Gene set enrichment analysis (GSEA, http://www.broadinstitute.org/gsea/index.jsp) was used to identify enrichments in specific signalling pathways between the LPS and LPS + TY-52156 groups. GSEA was performed with hallmark or KEGG gene set collections.

### Recombinant AAV-shRNA vectors

We packaged AAV constructs in the viral vectors of serotype-6 AAVs. One hundred microlitres of S1PR3 AAV-shRNA or Ctrl AAV-shRNA (1.0 × 10^11^ viral particles) was delivered intratracheally to the mice. Mice were used for experiments four weeks after AAV injection to allow sufficient time for viral transduction.

### Western blot

Proteins were extracted from lung tissues or cultured cells using a total protein extraction kit. A BCA protein quantification kit was used to determine equal amounts of protein added from each sample. Proteins were boiled with 4 × protein SDS‒PAGE loading buffer and subjected to 10% SDS‒PAGE separation gel (EpiZyme, Shanghai, China). Proteins resolved in SDS-PAGE were transferred to a 0.2 µm PVDF membrane (Millipore, MA, USA). The membranes were incubated with primary antibody at 4 °C overnight after blocking with 5% milk or BSA. The next day, the membranes were washed and incubated with an HRP-conjugated secondary antibody for 1 h at room temperature, and the signal was visualized using an enhanced chemiluminescence (ECL) kit. The immunological signal intensity was quantified using ImageJ software (National Institutes of Health). The following antibodies were used for the WB assays at the concentrations reported in parentheses: anti-S1PR3 (1:2500), anti-VE-cadherin (1:1000), anti-β-catenin (1:5000), anti-VCAM-1 (1:1000), anti-p-IKBα (1:3000), anti-IKBα (1:2000), anti-p-IKKβ (1:1000), anti-IKKβ (1:1000), anti-p-NF-κB (1:1000), anti-NF-κB (1:1000), anti-GAPDH (1:3000) and HRP goat anti-rabbit IgG (H + L) (1:5000) antibodies.

### ELISA

The levels of IL-6 and TNF-α in the serum, BALF, intracellular and cell supernatants were measured using the corresponding Quantikine ELISA kits. The assay was conducted according to the manufacturer's protocol.

### Cell culture

Human umbilical vein endothelial cells (HUVECs) were cultured in RPMI 1640 culture medium supplemented with 10% fetal bovine serum and 1% penicillin–streptomycin. The cells were incubated at 37 °C in 5% CO_2_ until they formed a confluent monolayer. Cells were pretreated with TY-52156 (10 μM) for 1 h before LPS administration and then exposed to LPS (10 μg/mL) or PBS in a serum-free medium for 48 h. For siRNA experiments, cells were pretreated with NF-κΒ activator 2 (10 μM) or oligomycin (5 μM) before LPS/PBS administration.

### Small interfering RNA (siRNA) transfection

HUVECs (2.0 × 10^5^ cells/well), seeded in a 12-well plate and grown to approximately 70% confluence, were transfected with 80 nM siRNA using Lipofectamine 3000 reagent for 48 h, and subsequent related experiments were performed.

### Scratch assay

Cells (1.0 × 10^6^ cells/well) were seeded into a 6-well plate and allowed to reach approximately 100% confluence. Then, we made wounds at the bottom of the wells using a 200 μL pipette tip and washed them with PBS to remove nonadherent cells. Relative cell migration in each group was evaluated 48 h post-wounding using an inverted microscope (CKX53 Olympus, Tokyo, Japan).

### Transwell assay

Transwell experiments were performed using 24-well (8 μm-diameter pores) Transwell inserts. The upper chamber was serum-free, while the lower chamber contained medium supplemented with 10% foetal bovine serum. Homogeneous single-cell suspensions (200 μL at 1 × 10^6^ cells/mL) were added to the upper chambers and allowed to invade for 48 h. Then, the migrated cells in each group were fixed, stained with crystal violet, and imaged using an inverted microscope (CKX53 Olympus, Tokyo, Japan).

### Cell immunofluorescence

HUVECs were fixed in 4% paraformaldehyde and blocked with 5% goat serum. The cells were then incubated overnight at 4 °C with the primary antibody diluted in 1% BSA/PBS. An Alexa Fluor 488-conjugated anti-rabbit IgG antibody was used as the secondary antibody, and the sections were further incubated for 1 h. F-Actin was stained with rhodamine-conjugated phalloidin (1:200 dilution) for 1 h. The cell nuclei were counterstained with DAPI for 10 min. All further incubations were performed at room temperature, and each incubation step was followed by three washes with PBS (5 min each).

### Flow cytometric analysis

Apoptotic cells were assessed by flow cytometric analysis using an Annexin V-FITC/PI apoptosis detection kit. Briefly, HUVECs (2.0 × 10^5^ cells) were trypsinized, centrifuged, and resuspended in 500 μL of diluted 1 × Annexin V binding buffer. Then, 3 μL of Annexin-V-FITC and PI staining reagents were added to the cell suspension. The solution was gently mixed and incubated for 15 min at room temperature in the dark. The percentage of apoptotic cells in each group was measured using a flow cytometer (Beckman Coulter).

### Cellular permeability assay

Endothelial permeability was assessed by measuring fluorescein isothiocyanate (FITC)- conjugated dextran (average MW: 10000) across the HUVEC monolayer [22]. Briefly, HUVECs (200 μL at 1.0 × 10^6^ cells/mL) were seeded into the upper chamber to form a confluent monolayer and subjected to different treatments. Then, FITC-dextran (1 mg/mL) was added to the upper chamber and incubated for 30 min at 37 °C. The fluorescence of FITC-dextran was measured using a plate reader at excitation/emission wavelengths of 490/520 nm.

### DHE staining

DHE staining was performed to evaluate ROS production in the lung tissues. Frozen lung tissue Sects. (8 μm) were incubated with 5 μM DHE solution for 30 min at 37 °C in the dark.

### Mitochondrial ROS (mtROS) assay

Cells were seeded into 6-well plates at a density of 8.0 × 10^5^ cells/well. Mito-SOX Red was used to detect mtROS in HUVECs. The cells were incubated with 5 μM fluorochrome at 37 °C for 30 min and washed with PBS, and Hoechst was used to stain the cell nuclei for 5 min.

### Mitochondrial membrane potential

Cells were seeded into 6-well plates at a density of 8.0 × 10^5^ cells/well. The mitochondrial membrane potential detection was determined using a JC-1 kit. The cells were incubated with 2 μM JC-1 at 37 °C for 20 min, and washed with PBS, and Hoechst was used to stain the cell nuclei for 5 min. Fluorescence microscopy was used to measure the ratio of green (JC-1 monomers)/red (JC-1 aggregates) fluorescence.

### ATP detection

The ATP level was determined using an ATP assay kit. The final ATP content was normalized to its protein concentration measured by a BCA assay.

### Activity of the mitochondrial respiratory complex

Mitochondrial respiratory chain complex activities were measured according to the kit instructions.

### Statistical analysis

Statistical analysis and graph creation were performed using GraphPad Prism 9.0 software (San Diego, CA, USA). Multiple group comparisons were analyzed with one-way ANOVA followed by Turkey post-hoc test. All tests were two-sided, with significance set at *p* ≤ 0.05.

## Results

### S1PR3 expression was increased over time after LPS stimulation

**Fig. 1:**
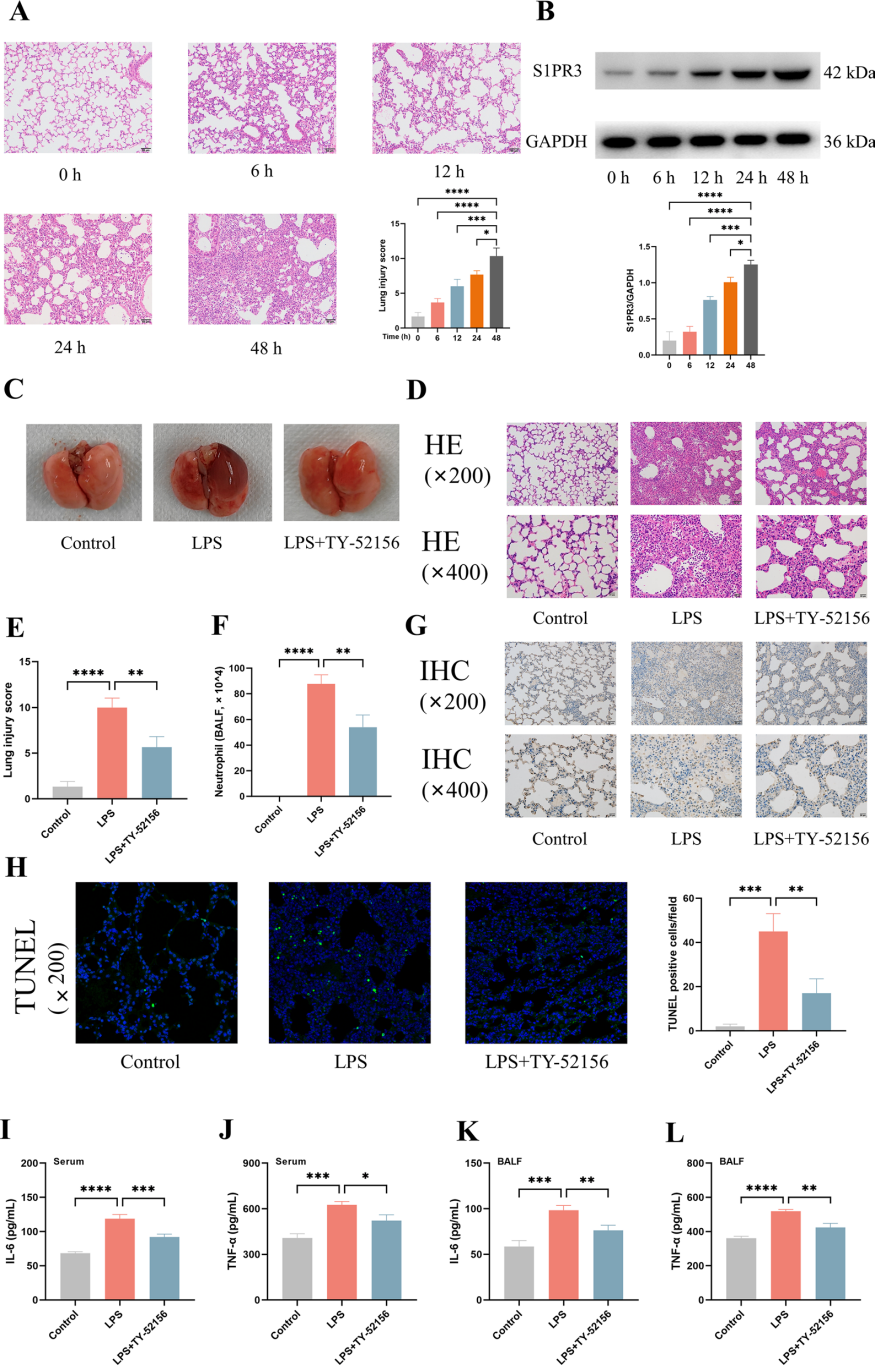
Time course protein S1PR3 changes in lung tissues after LPS stimulation, and TY-52156 alleviated LPS-induced lung inflammation in mice. Mice were intratracheally injected with LPS (5 mg/kg) or PBS, and the lung tissues were harvested at different time points (0, 6, 12, 24 and 48 h) after LPS stimulation. **A** H&E staining (× 200) of the lung tissues (n = 3 for each group). **B** The expression of S1PR3 was detected via western blot analysis in lung tissues (n = 3 for each group). Protein bands were quantified using densitometry, and their relative values were expressed normalized to GAPDH band. Mice were intratracheally injected with LPS (5 mg/kg) or PBS, and TY-52156 (10 mg/kg) was injected intraperitoneally 1 h before LPS injection. Lung tissues were harvested at 48 h after LPS stimulation. **C** Gross picture of the lungs (n = 3 for each group). **D**, **E** H&E staining (D; × 200 and × 400) and corresponding lung injury score (**E**). **F** Neutrophil counts in BALF (n = 3 for each group). **G** Immunohistochemical staining (× 200 and × 400) of Ly6G in lung tissues (n = 3 for each group). **H** TUNEL fluorescence staining (× 200) of lung tissues (n = 3 for each group). **I**, **J** The concentration of IL-6 (**I**) and TNF-α (**J**) in serum of mice (n = 3 for each group). **K**, **L** The concentration of IL-6 (**K**) and TNF-α (**L**) in BALF of mice (n = 3 for each group). The data are presented as the mean ± S.D. Significant differences are shown by ^∗^*p* < 0.05, ^∗∗^*p* < 0.01, ^∗∗∗^*p* < 0.001, and ^∗∗∗∗^*p* < 0.0001

Previous studies have revealed that S1PR3 was increased in patients with ARDS [14–16], and we further examined the expression of S1PR3 in the lung tissues of mice with LPS-induced ARDS. In the present study, mice were intratracheally injected with LPS to model bacterium-induced ARDS. Lung tissue samples were harvested at different time points (0, 6, 12, 24 and 48 h) after LPS stimulation, and H&E staining was performed. We found that lung tissue damage increased over time, and the typical histopathological changes of acute lung injury e.g., alveolar thickening, interstitial oedema and inflammatory cell infiltration, were observed (Fig. 1A). Western blot analysis revealed that S1PR3 expression was increased in the lung tissues over time (Fig. 1B). These findings indicate that S1PR3 expression is positively correlated with the severity of acute lung injury.

### TY-52156 alleviated inflammation in mouse lung tissues and HUVECs.

The significant upregulation of S1PR3 suggested that S1PR3 might be involved in the pathogenesis of ARDS. To assess the effects of S1PR3 inhibition on LPS-induced acute lung injury, we identified the gross pathological and histopathological changes in the lung tissues. We found that the LPS-injected mice exhibited severe congestion and oedema in the lungs, whereas the lungs of the mice pretreated with TY-52156 exhibited fewer changes, as mentioned above (Fig. 1C). Histologically, tissue damage was milder in the LPS + TY-52156 group than that in the LPS group (Fig. 1D). The lung injury score increased from 1.3 ± 0.58 in the control group to 10.00 ± 1.00 in the LPS group and decreased to 5.67 ± 1.16 in the LPS + TY-52156 group (Fig. 1E). The inflammatory response is closely related to the progression of acute lung injury, and the accumulation of neutrophils in the lungs is a well-documented feature of pulmonary inflammation [25]. As shown in Fig. 1F, the number of BALF neutrophils in the LPS + TY-52156 group was significantly lower than that in the LPS group. IHC analysis of the neutrophil-specific marker Ly6G also revealed that the neutrophil infiltration in the LPS + TY-52156 group was reduced compared with that in the LPS group (Fig. 1G). Moreover, we found that LPS increased the number of TUNEL-positive cells in mice, whereas the increase in the number of apoptotic cells was attenuated by TY-52156 treatment (Fig. 1H). Proinflammatory cytokines are quantitative markers of inflammation [25], so we measured the levels of two proinflammatory cytokines (IL-6 and TNF-α) in the serum and BALF. We found that the levels of IL-6 and TNF-α were increased after LPS stimulation, whereas the levels of these cytokines were significantly decreased in the group pretreated with TY-52156 before LPS stimulation (Fig. 1I-L). Thus, TY-52156 alleviated LPS-induced lung inflammation in mice.

**Fig. 2:**
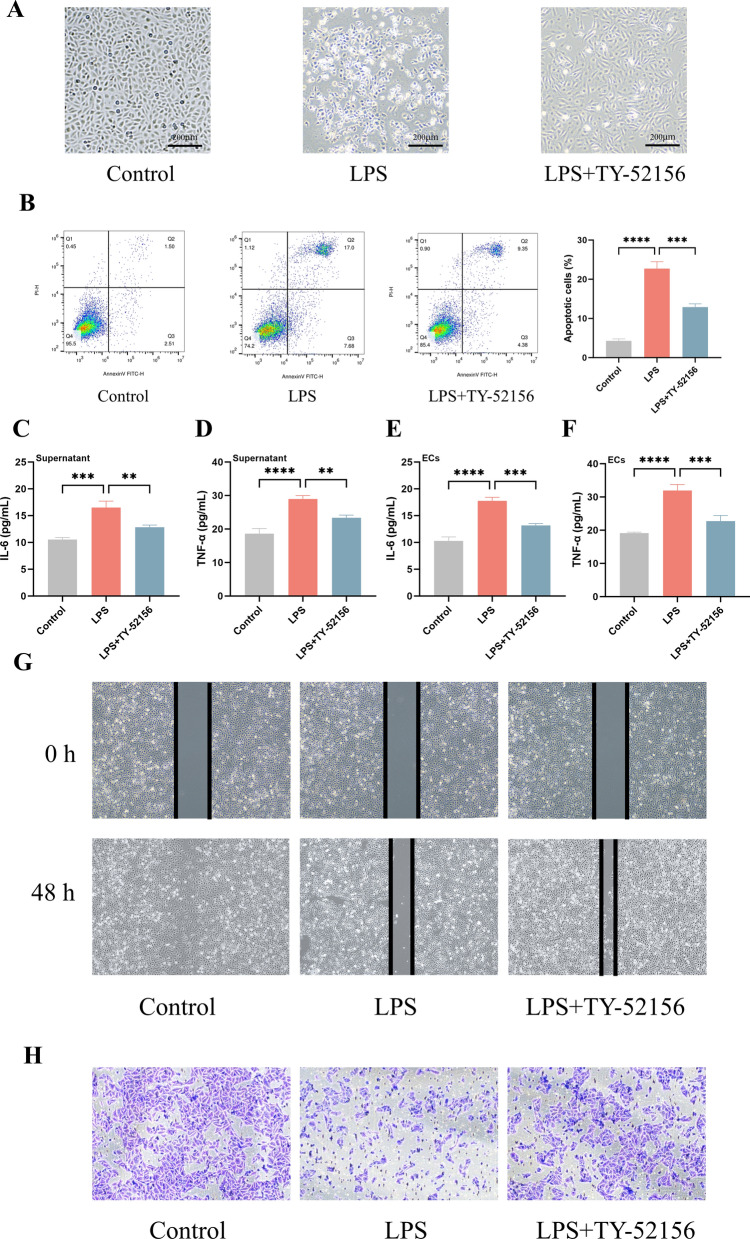
TY-52156 alleviated LPS-induced inflammation in HUVECs**.** ECs were pretreated with TY-52156 (10 μM) for 1 h before LPS administration and then exposed to LPS (10 μg/mL) or PBS in a serum-free medium for 48 h. **A** White light image (× 40) of the cell (n = 3 for each group). **B** Flow cytometric analysis of cell apoptosis (n = 3 for each group). **C**, **D** The concentration of IL-6 (**C**) and TNF-α (**D**) in cell supernatant (n = 3 for each group). **E**, **F** The concentration of IL-6 (**E**) and TNF-α (**F**) in ECs (n = 3 for each group).** G** Cell scratch assay images (× 40) at 0 h and 48 h (n = 3 for each group). **H** Images of cell transwell invasion assay (× 100) after 48 h incubation (n = 3 for each group). The data are presented as the mean ± S.D. Significant differences are shown by ^∗^*p* < 0.05, ^∗∗^*p* < 0.01, ^∗∗∗^*p* < 0.001, and ^∗∗∗∗^*p* < 0.0001

Next, we examined whether TY-52156 alleviates inflammation in ECs. LPS stimulation altered cell morphology and induced overt cell death, whereas pretreatment of cells with TY-52156 prevented this effect (Fig. 2A). Flow cytometric analysis revealed that TY-52156 significantly attenuated the LPS-induced increase in the percentage of apoptotic cells (Fig. 2B). Similar to the in vivo experiments, TY-52156 significantly decreased the IL-6 and TNF-α levels in the supernatant and cell contents after LPS stimulation (Fig. 2C-F). We also evaluated the invasion and migration of ECs in different groups. Scratch assays showed that the cells in the LPS group healed more slowly than those in the control group, while the cells in the LPS + TY-52156 group healed faster than did those in the LPS group (Fig. 2G). Transwell assays showed that TY-52156 increased the number of transmembrane cells (Fig. 2F). These data indicate that TY-52156 has an anti-inflammatory effect on ECs.

### TY-52156 alleviated LPS-induced endothelial barrier injury in mice and in HUVECs

**Fig. 3:**
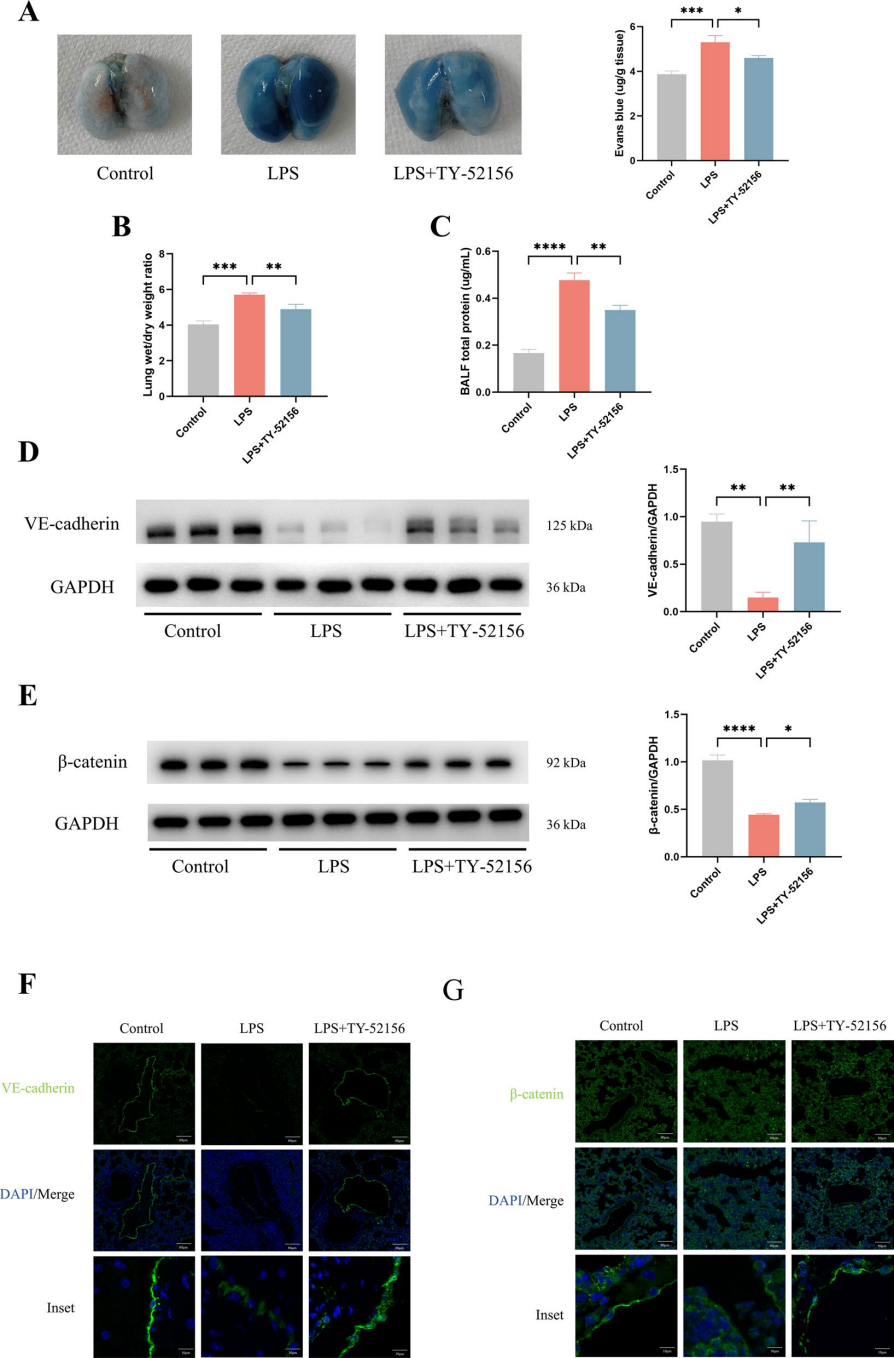
TY-52156 alleviated LPS-induced endothelial barrier injury in mice**.** Mice were intratracheally injected with LPS (5 mg/kg) or PBS, and TY-52156 (10 mg/kg) was injected intraperitoneally 1 h before LPS injection. Lung tissues were harvested at 48 h after LPS stimulation. **A** Evans blue dye analysis of lung tissues (n = 3 for each group). **B** Lung wet/dry weight ratio (n = 3 for each group). **C** BALF total protein levels (n = 3 for each group). **D**, **E** The expression of VE-cadherin (**D**) and β-catenin (**E**) were detected via western blot analysis in lung tissues (n = 3 for each group). Protein bands were quantified using densitometry, and their relative values were expressed normalized to GAPDH band. **F**, **G** Immunofluorescence staining (× 100) of VE-cadherin (**F**) and β-catenin (**G**) in lung tissues (n = 3 for each group). The data are presented as the mean ± S.D. Significant differences are shown by ^∗^*p* < 0.05, ^∗∗^*p* < 0.01, ^∗∗∗^*p* < 0.001, and ^∗∗∗∗^*p* < 0.0001

Endothelial barrier dysfunction leads to a significant increase in cell permeability, which is a key pathological feature of ARDS [6]. Our study suggested that LPS injection significantly increased murine pulmonary vascular permeability (Evans blue concentration, Fig. 3A), lung oedema (lung W/D, Fig. 3B) and BALF total protein levels (Fig. 3C). The expression of adherens junctions (AJs) protein (VE-cadherin and β-catenin), as detected by WB and immunofluorescence staining, decreased after LPS stimulation. The administration of TY-52156 significantly prevented these changes (Fig. 3D–G). These data indicate that TY-52156 protected the pulmonary endothelial barrier in vivo.

**Fig. 4:**
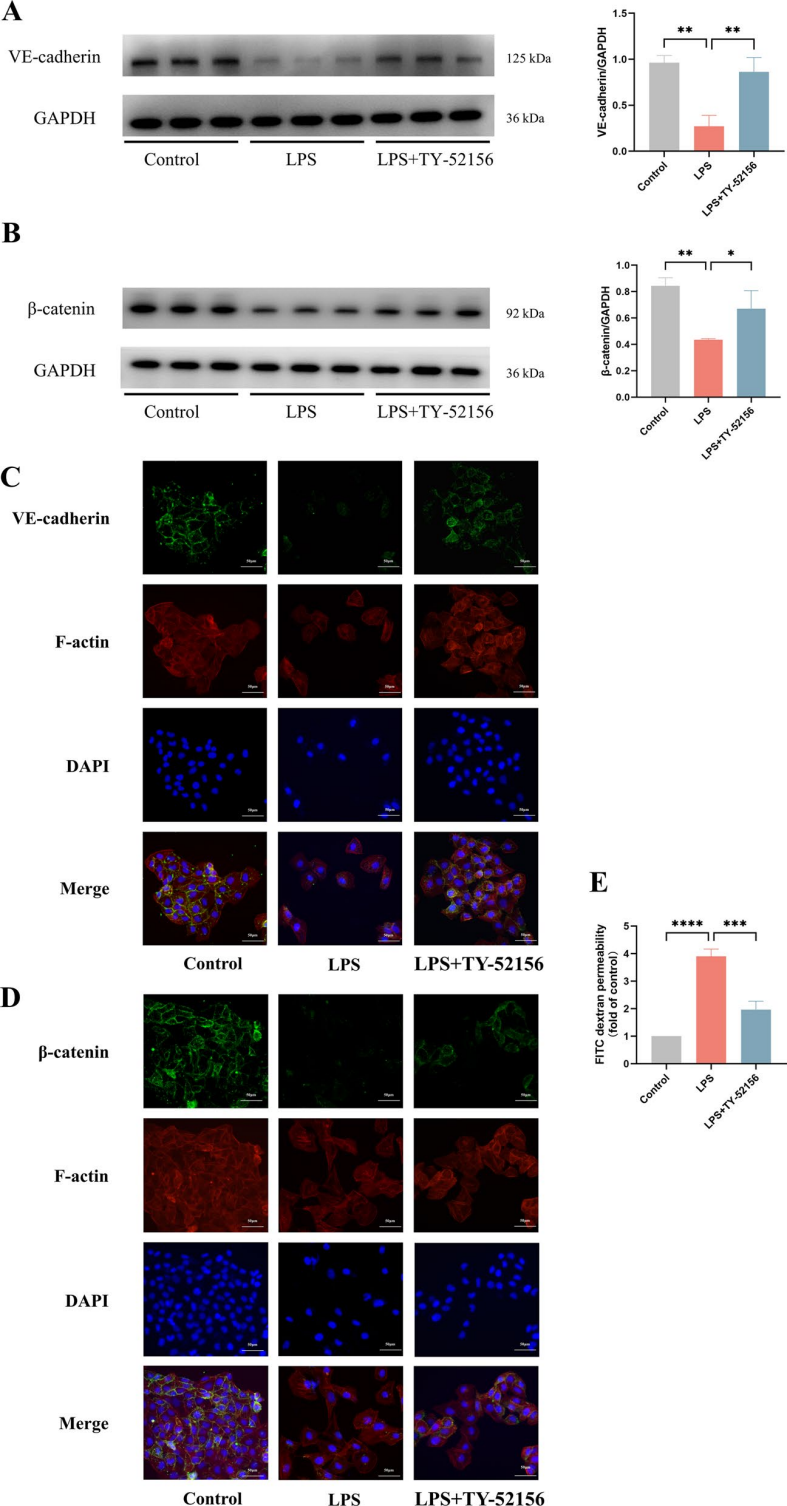
TY-52156 alleviated LPS-induced endothelial barrier injury in HUVECs. ECs were pretreated with TY-52156 (10 μM) for 1 h before LPS administration and then exposed to LPS (10 μg/mL) or PBS in a serum-free medium for 48 h. **A**, **B** The expression of VE-cadherin (**A**) and β-catenin (**B**) were detected via western blot analysis in ECs. Protein bands were quantified using densitometry, and their relative values were expressed normalized to GAPDH band (n = 3 for each group). **C**, **D** Immunohistochemical staining (× 200) of VE-cadherin (**C**) and β-catenin (**D**) in ECs (n = 3 for each group). **E** FITC-dextran cellular permeability assay with ECs (n = 3 for each group). The data are presented as the mean ± S.D. Significant differences are shown by ^∗^*p* < 0.05, ^∗∗^*p* < 0.01, ^∗∗∗^*p* < 0.001, and ^∗∗∗∗^*p* < 0.0001

Next, we investigated whether TY-52156 can alleviate endothelial barrier injury in ECs. The AJs and actin cytoskeleton are important for the stabilization of the pulmonary EC barrier [26]. We therefore measured the expression of AJs by WB and immunofluorescence staining and the assembly of the actin cytoskeleton by phalloidin staining to assess the effects of TY-52156 on vascular homeostasis. As expected, our results suggested that the expressions of VE-cadherin and β-catenin were significantly decreased after LPS stimulation (Fig. 4A–D). Phalloidin staining revealed F-actin reorganization and stress fibre formation in the LPS-induced ECs (Fig. 4C, D). TY-52156 administration reversed the deleterious effects of LPS on ECs. The influx of FITC-dextran revealed that TY-52156 reduced the LPS-induced hyperpermeability of ECs (Fig. 4E), indicating the protective effect of this treatment on the endothelial barrier.

### Transcriptomic profiling of lung tissues from the mice treated with LPS or LPS + TY-52156.

**Fig. 5:**
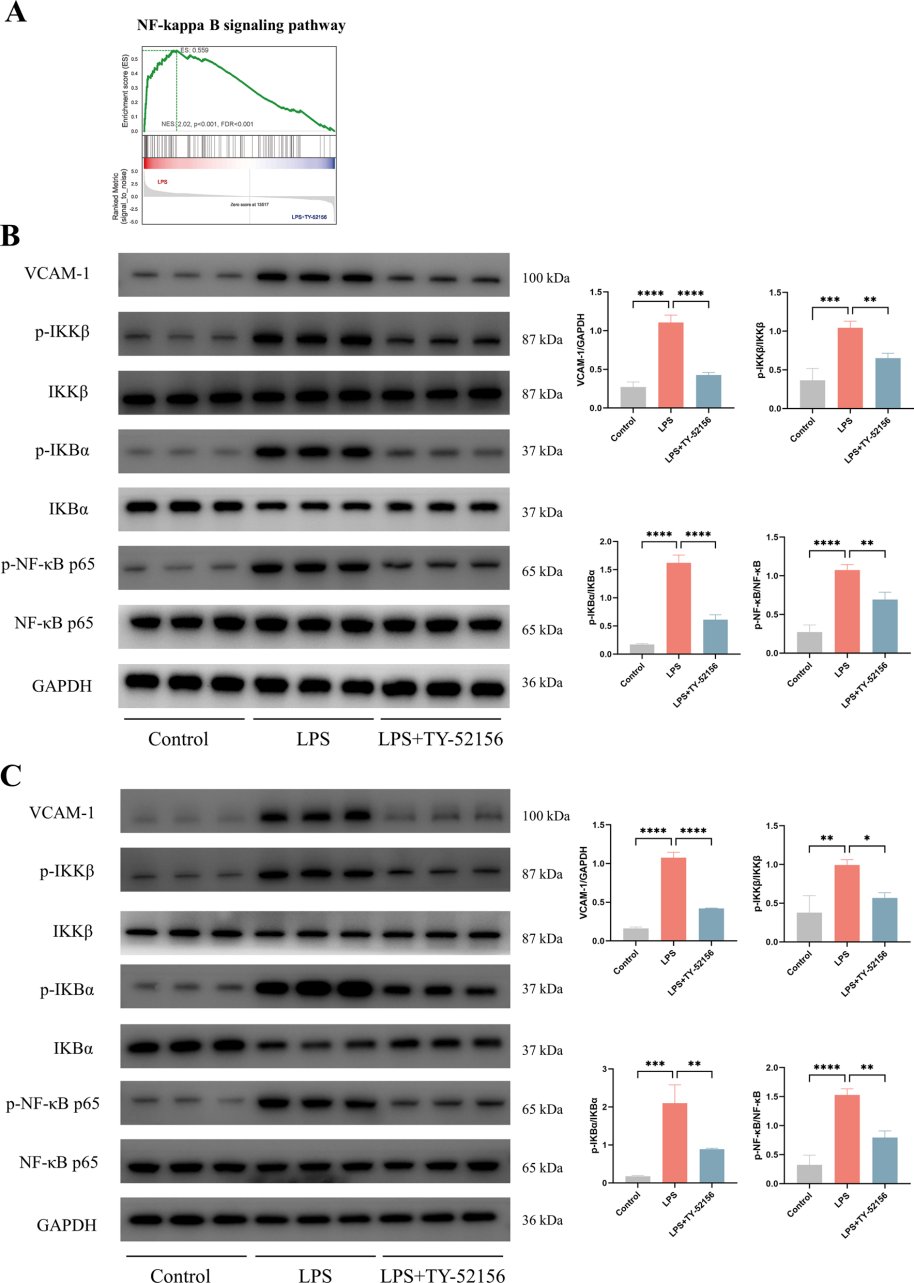
TY-52156 downregulated NF-κB pathway in mice and in HVUECs.** A** GSEA analysis of the ‘NF-κB signaling’ pathway between LPS group and LPS + TY-52156 group. **B, C** Western blot analysis of VCAM-1, p-IKKβ, IKKβ, p-IKBα, IKBα, p-NF-κB, NF-κB and GAPDH in lung tissues (**B**) and ECs (**C**) (n = 3 for each group). Protein bands were quantified using densitometry. The relative phosphorylation levels of protein were expressed normalized to the total protein band, and others were expressed normalized to GAPDH band. The data are presented as the mean ± S.D. Significant differences are shown by ^∗^*p* < 0.05, ^∗∗^*p* < 0.01, ^∗∗∗^*p* < 0.001, and ^∗∗∗∗^*p* < 0.0001

**Fig. 6:**
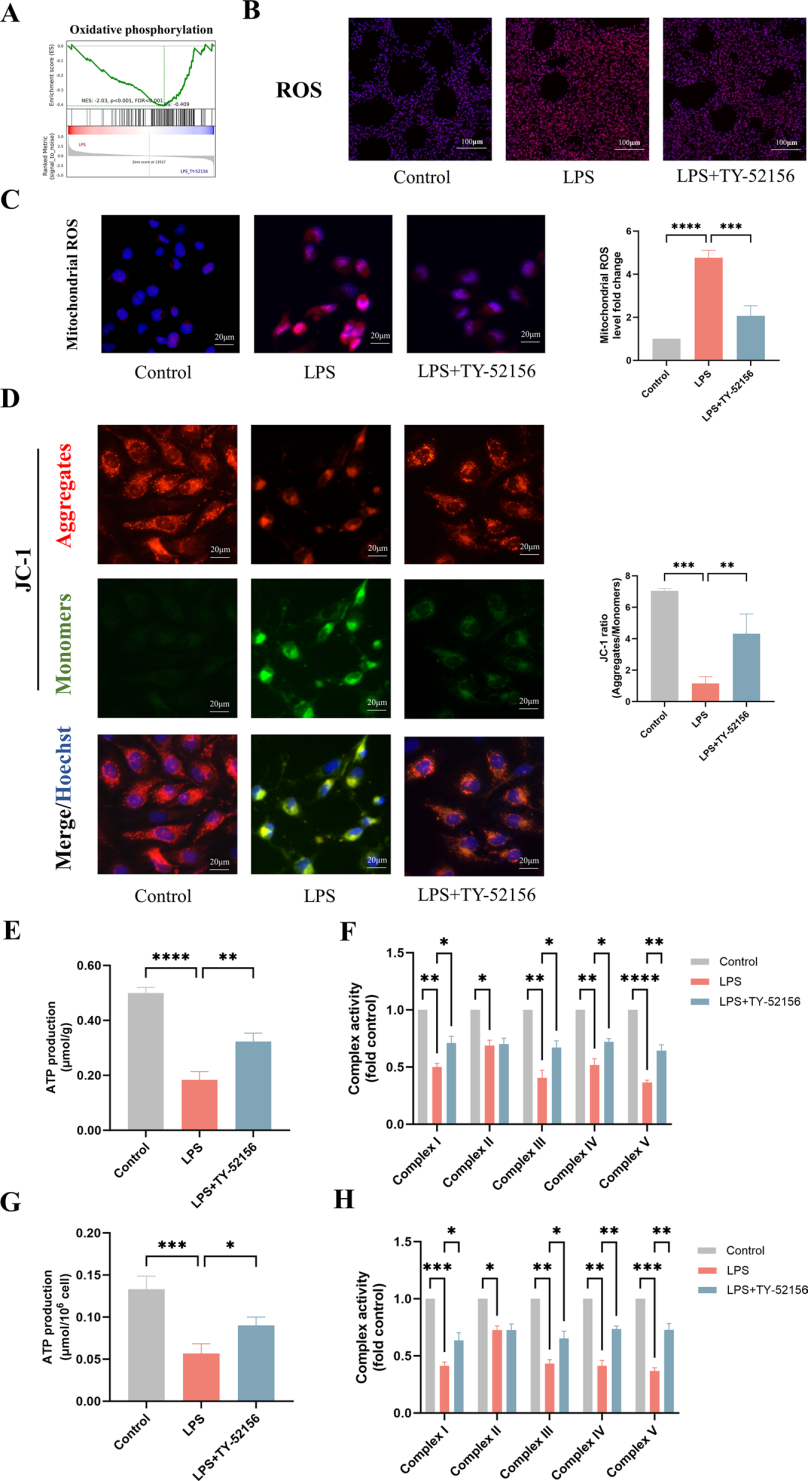
TY-52156 activated mitochondrial oxidative phosphorylation in mice and in HUVECs.** A** GSEA analysis of the ‘Oxidative phosphorylation’ pathway between LPS group and LPS + TY-52156 group. **B** DHE staining (× 100) to evaluate ROS level in lung tissues (n = 3 for each group). **C** Detection of mitochondrial ROS production (× 200) in ECs (n = 3 for each group). **D** Detection of mitochondrial membrane potential (× 200) by JC‐1 in ECs (n = 3 for each group). **E** Detection of ATP production in lung tissues (n = 3 for each group). **F** Detection of mitochondrial complex I-IV activity in lung tissues (n = 3 for each group). **G** Detection of ATP production in ECs (n = 3 for each group). **G**. Detection of mitochondrial complex I-IV activity in ECs (n = 3 for each group). The data are presented as the mean ± S.D. Significant differences are shown by ^∗^*p* < 0.05, ^∗∗^*p* < 0.01, ^∗∗∗^*p* < 0.001, and ^∗∗∗∗^*p* < 0.0001

To investigate the mechanism by which S1PR3 inhibition prevents LPS-induced ARDS, we examined the S1PR3-mediated effects at transcriptional level. Heatmap analysis of the transcriptome data indicated a clear difference between the transcriptome profiles of the LPS and LPS + TY-52156 groups (Additional file 1: Fig. S1A). The GO and KEGG analyses suggested that differentially expressed genes were enriched in the inflammatory response, cell adhesion molecules and cytokine‒cytokine receptor interactions (Additional file 1: Fig. S1B, C). Because the NF- κB signalling pathway and mitochondrial oxidative stress are closely associated with these functions [28, 29], we used GSEA to test these relevant signalling pathways. GSEA revealed that the hallmark pathways for the inflammatory response (Additional file 1: Fig. S1D) and TNF-α signalling via NF-κB (Additional file 1: Fig. S1E) and the KEGG pathway for NF-κB (Fig. 5A) were significantly more highly activated in the LPS group than in the LPS + TY-52156 group. Moreover, compared to those in the LPS group, the expression of genes in the hallmark pathways for oxidative phosphorylation was significantly greater in the LPS + TY-52156 group (Fig. 6A).

### TY-52156 inhibited the NF-κB pathway both in vivo and in vitro.

To investigate whether TY-52156 affects the NF‐κB pathway, we examined the total and phosphorylated levels of p65, IKBα and IKKβ and the level of VCAM-1 via WB analysis. We found that the levels of p-p65-NF‐κB/p65-NF‐κB, p-IκBα/IκBα, p-IKKβ/IKKβ and VCAM-1/GAPDH were significantly increased after LPS stimulation, but were significantly decreased in the LPS + TY-52156 group (Fig. 5A, B).

### TY-52156 activated mitochondrial oxidative phosphorylation both in vivo and in vitro

To assess mitochondrial oxidative phosphorylation, we tested the levels of ROS, JC-1, ATP and mitochondrial complex I-V activity in the different groups. In the LPS group, the stained lung tissues displayed strong red fluorescence produced by DHE oxidation; nevertheless, the red fluorescence was reduced in the LPS + TY-52156 group (Fig. 6B). The LPS-induced increase in mitochondrial ROS levels in ECs was significantly suppressed by TY-52156 (Fig. 6C). Then, we assessed the mitochondrial membrane potential (MMP) using JC-1 staining, and mitochondrial depolarization led to the fluorescence changes from red to green. Our results suggested that TY-52156 restored the decrease in the MMP caused by LPS stimulation, as indicated by an increase in red fluorescence (JC-1 aggregates) and a decrease in green fluorescence (JC-1 monomers) (Fig. 6D).Compared with the control group, the ATP production and mitochondrial complex I-V activity were significantly decreased in the LPS group. However, TY-52156 treatment increased the levels of ATP production and mitochondrial complex I, III-V activity, compared with the LPS group (Fig. 6E–H).

### Inhibition of S1PR3 by AAV-S1PR3 in vivo and by siRNA-S1PR3 in vitro inhibited the NF-κB pathway

**Fig. 7:**
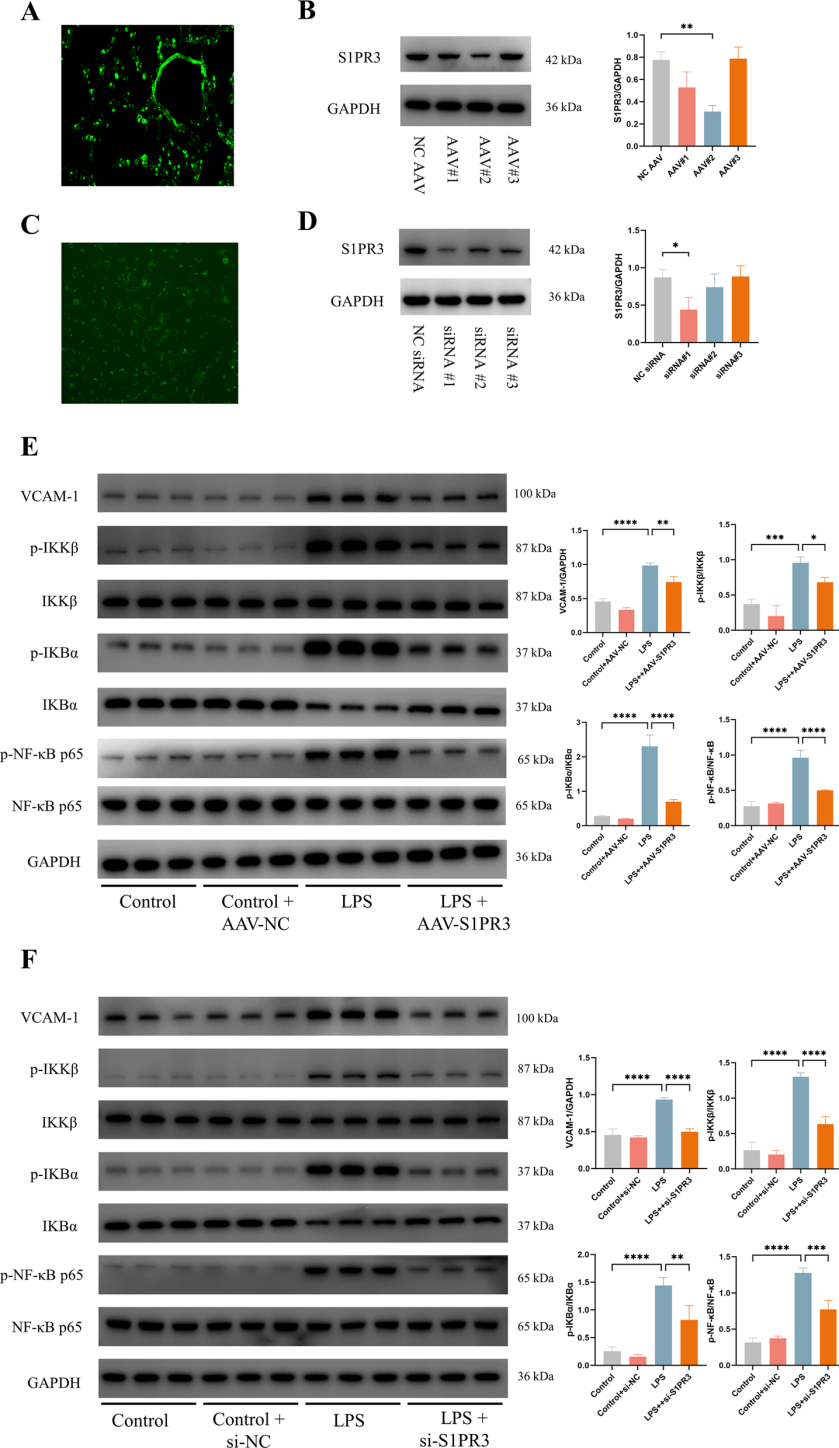
AAV-S1PR3 and si-S1PR3 downregulated NF-κB pathway in mice and in HUVECs, respectively. **A** Fluorescent image of AAV transfection in lung tissues (n = 3 for each group). **B** The expression of S1PR3 was detected via western blot analysis in lung tissues after AAV injection (n = 3 for each group). Protein bands were quantified using densitometry, and their relative values were expressed normalized to GAPDH band. **C** Fluorescent image of si-RNA transfection in ECs (n = 3 for each group). **D** The expression of S1PR3 was detected via western blot analysis in ECs after siRNA transfection (n = 3 for each group). Protein bands were quantified using densitometry, and their relative values were expressed normalized to GAPDH band. **E**, **F** Western blot analysis of VCAM-1, p-IKKβ, IKKβ, p-IKBα, IKBα, p-NF-κB, NF-κB and GAPDH in lung tissues (**E**) and ECs (**F**) (n = 3 for each group). Protein bands were quantified using densitometry. The relative phosphorylation levels of protein were expressed normalized to the total protein band, and others were expressed normalized to GAPDH band. The data are presented as the mean ± S.D. Significant differences are shown by ^∗^*p* < 0.05, ^∗∗^*p* < 0.01, ^∗∗∗^*p* < 0.001, and ^∗∗∗∗^*p* < 0.0001

Because TY-52156 is a selective inhibitor of S1PR3, we used AAV and siRNA to inhibit the expression of S1PR3 to further verify our findings. In vivo, green fluorescence was detected in lung tissues four weeks after delivery, indicating successful AAV transfection (Fig. 7A). WB analysis revealed that the S1PR3 protein expression decreased significantly with the transduction of S1PR3 AAV#2 (Fig. 7B); hence, this construct was selected for subsequent experiments. In vitro, ECs were successfully transfected with siRNA (Fig. 7C), and S1PR3 siRNA#1 had the greatest inhibitory effect on S1PR3 expression (Fig. 7D). As expected, we found that inhibition of S1PR3 significantly reduced the levels of p-p65-NF‐κB/p65-NF‐κB, p-IκBα/IκBα, p-IKKβ/IKKβ and VCAM-1/GAPDH induced by LPS stimulation both in vivo and in vitro (Fig. 7E, F).

### Inhibition of S1PR3 by AAV-S1PR3 in vivo and by siRNA-S1PR3 in vitro activated mitochondrial oxidative phosphorylation

**Fig. 8:**
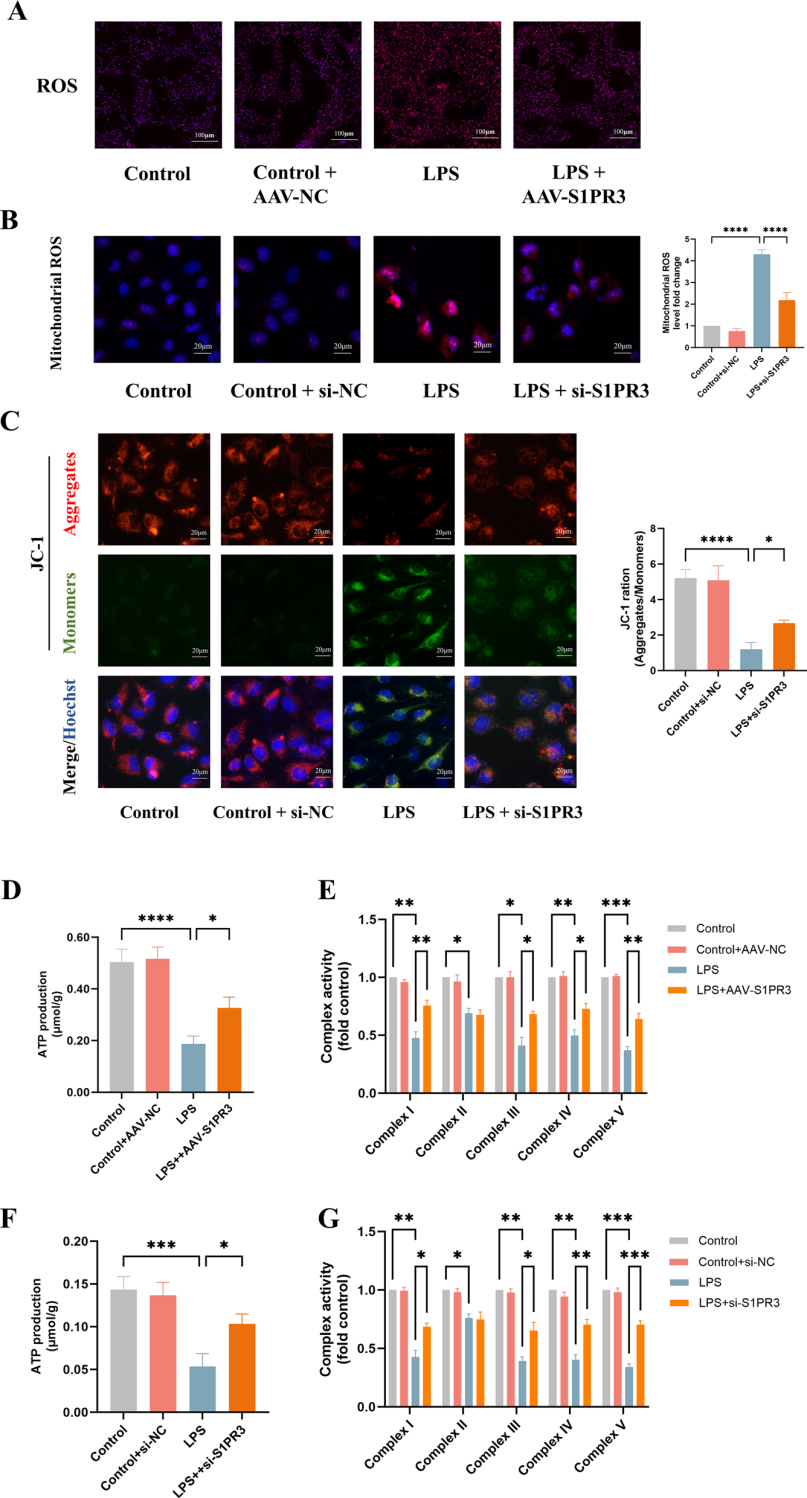
AAV-S1PR3 and si-S1PR3 activated mitochondrial oxidative phosphorylation in mice and in HUVECs, respectively. **A** DHE staining (× 100) to evaluate ROS level in lung tissues (n = 3 for each group).** B** Detection of mitochondrial ROS production (× 200) in ECs (n = 3 for each group). **C** Detection of mitochondrial membrane potential (× 200) by JC‐1 in ECs (n = 3 for each group). **D** Detection of ATP production in lung tissues (n = 3 for each group). **E** Detection of mitochondrial complex I-IV activity in lung tissues (n = 3 for each group). **F** Detection of ATP production in ECs (n = 3 for each group). **G** Detection of mitochondrial complex I-IV activity in ECs (n = 3 for each group). The data are presented as the mean ± S.D. Significant differences are shown by ^∗^*p* < 0.05, ^∗∗^*p* < 0.01, ^∗∗∗^*p* < 0.001, and ^∗∗∗∗^*p* < 0.0001

We then examined the effect of S1PR3 inhibition on mitochondrial oxidative phosphorylation in vivo and in vitro. The fluorescence intensity of DHE in lung tissues and mitochondrial ROS in ECs were reduced by S1PR3 inhibition after LPS stimulation (Fig. 8A, B). In addition, S1PR3 inhibition reversed the LPS-induced decrease in the MMP in ECs (Fig. 8C). S1PR3 inhibition also increased the ATP production and mitochondrial complex I, III-V activity induced by LPS stimulation both in vivo and in vitro (Fig. 8D–G).

### Activation of the NF-κB pathway suppressed the protective effect of S1PR3 inhibition on LPS-induced ARDS

**Fig. 9:**
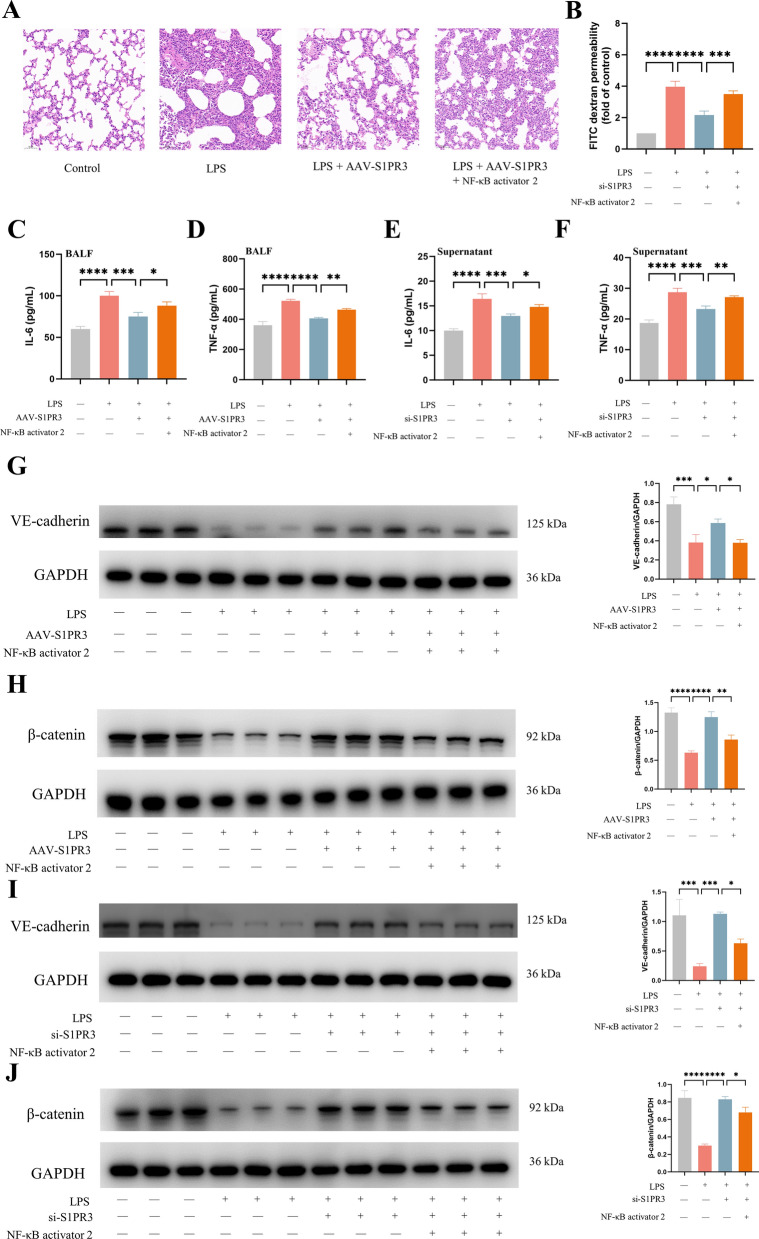
NF-κB activation weakened S1PR3 inhibition's protective effect against LPS-induced inflammation and endothelial barrier injury. **A** H&E staining (× 200) of the lung tissues (n = 3 for each group). B FITC-dextran cellular permeability assay with ECs (n = 3 for each group). **C**, **D** The concentration of IL-6 (**C**) and TNF-α (**D**) in BALF (n = 3 for each group). **E**, **F** The concentration of IL-6 (**E**) and TNF-α (**F**) in cell supernatant. G, H The expression of VE-cadherin (**G**) and β-catenin (**H**) were detected via western blot analysis in lung tissues (n = 3 for each group). Protein bands were quantified using densitometry, and their relative values were expressed normalized to GAPDH band. I, J The expression of VE-cadherin (**I**) and β-catenin (**J**) were detected via western blot analysis in ECs (n = 3 for each group). Protein bands were quantified using densitometry, and their relative values were expressed normalized to GAPDH band. The data are presented as the mean ± S.D. Significant differences are shown by ^∗^*p* < 0.05, ^∗∗^*p* < 0.01, ^∗∗∗^*p* < 0.001, and ^∗∗∗∗^*p* < 0.0001.

To explore the role of the NF-κB pathway in S1PR3 inhibition-mediated protection against LPS-induced ARDS, we used NF-κB activator 2 before LPS stimulation in AAV-S1PR3 mice and in siRNA-S1PR3 ECs. We found that NF-κB activator 2 increased the degree of lung injury in mice (Fig. 9A) and increased FITC dextran permeability in ECs (Fig. 9B), increased the levels of IL-6 and TNF-α in BALF and cell supernatant (Fig. 9C–F), and decreased the expression of VE-cadherin and β-catenin both in vivo and in vitro (Fig. 9G–J).

### Inhibition of mitochondrial oxidative phosphorylation suppressed the protective effect of S1PR3 inhibition on LPS-induced ARDS

**Fig. 10:**
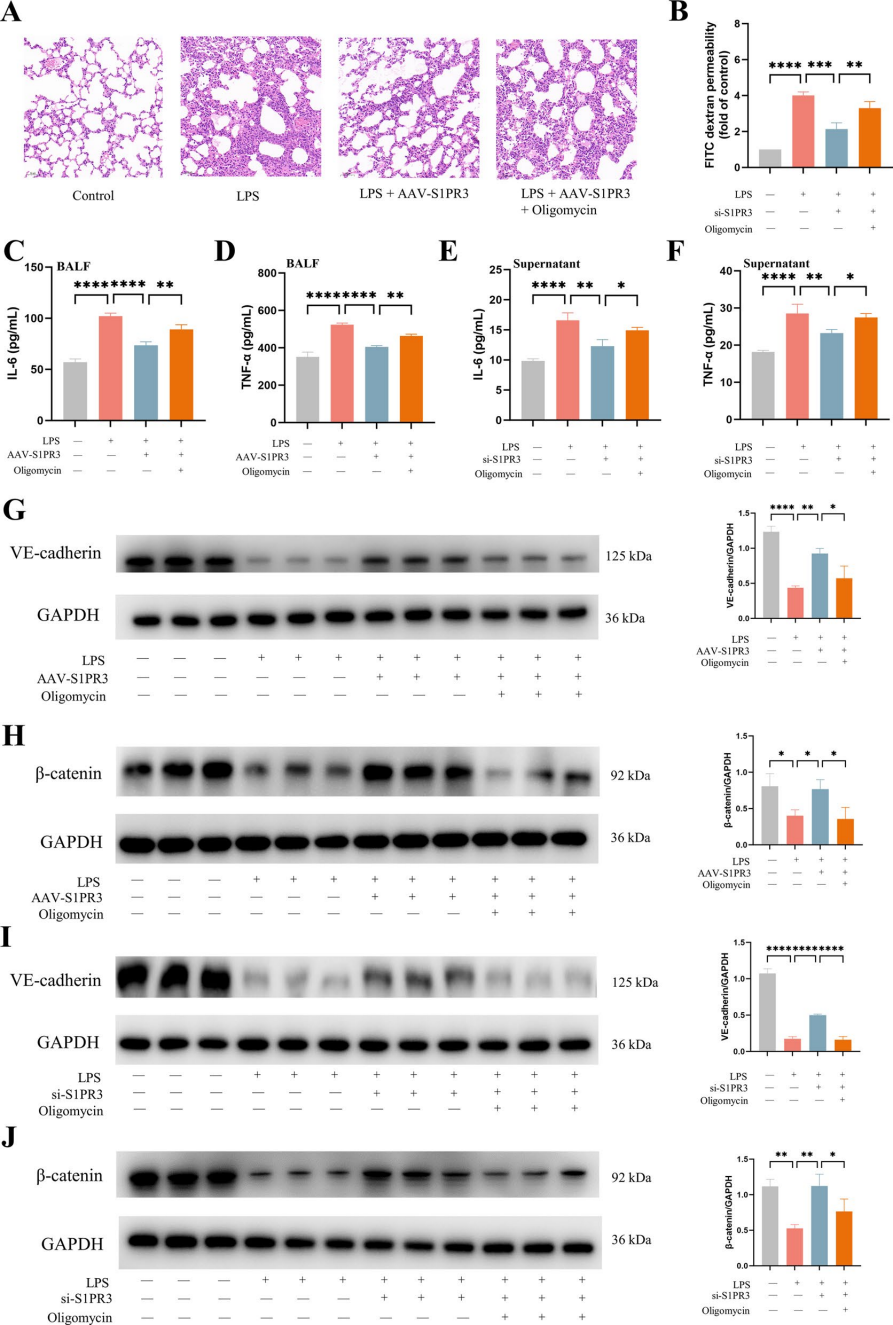
OXPHOS inhibition weakened S1PR3 inhibition's protective effect against LPS-induced inflammation and endothelial barrier injury. **A** H&E staining (× 200) of the lung tissues (n = 3 for each group). **B** FITC-dextran cellular permeability assay with ECs (n = 3 for each group). **C, D** The concentration of IL-6 (**C**) and TNF-α (**D**) in BALF (n = 3 for each group). **E**, **F** The concentration of IL-6 (**E**) and TNF-α (**F**) in cell supernatant (n = 3 for each group). **G**, **H** The expression of VE-cadherin (**G**) and β-catenin (**H**) were detected via western blot analysis in lung tissues (n = 3 for each group). Protein bands were quantified using densitometry, and their relative values were expressed normalized to GAPDH band. **I**, **J** The expression of VE-cadherin (**I**) and β-catenin (**J**) were detected via western blot analysis in ECs (n = 3 for each group). Protein bands were quantified using densitometry, and their relative values were expressed normalized to GAPDH band. The data are presented as the mean ± S.D. Significant differences are shown by ^∗^*p* < 0.05, ^∗∗^*p* < 0.01, ^∗∗∗^*p* < 0.001, and ^∗∗∗∗^*p* < 0.0001

We next explored the role of mitochondrial oxidative phosphorylation in S1PR3 inhibition-mediated protection against LPS-induced ARDS. Oligomycin, an inhibitor of mitochondrial oxidative phosphorylation, was used in the present study. We found that oligomycin increased the degree of lung injury in mice (Fig. 10A), increased the FITC dextran permeability in ECs (Fig. 10B), increased the levels of IL-6 and TNF-α in BALF and cell supernatant (Fig. 10C–F), and decreased the expression of VE-cadherin and β-catenin in vivo and in vitro (Fig. 10G–J).

## Discussion

Our study suggested a critical role of S1PR3 in the regulation of the inflammatory response and endothelial barrier function in ARDS. S1PR3 is highly expressed in lung tissues and the endothelium. S1PR3 has been widely described as a mediator of various biological responses [30], and the exact roles of S1PR3 vary in different cells, tissues and diseases under different conditions. Wang et al. [31] reported that S1PR3 inhibition suppressed the activation of the NLPR3 inflammasome in LPS-stimulated macrophages as well as in the lungs of septic mice. Bajwa et al. [32] reported that S1PR3-deficient dendritic cells have a protective effect on ischaemia–reperfusion injury-induced acute kidney injury. However, the relationship between S1PR3 and ARDS is still unclear. Our results suggested that S1PR3 expression was increased in the lungs of LPS-induced ARDS mice, and S1PR3 inhibition alleviated inflammation and endothelial injury in vivo and in vitro, thus reducing LPS-induced ARDS.

In response to lung injury, increased numbers of leukocytes, especially neutrophils, fold into the alveolar interstitium or alveolar space, which is known as the exudative phase [33]. The activation of neutrophils leads to the production of proinflammatory cytokines, such as IL-6 and TNF-α [34]. These proinflammatory cytokines increase the disruption of alveolar-capillary barriers, resulting in pulmonary oedema [1]. Thus, effective suppression of the inflammatory response is an important treatment strategy for ARDS. Our present study revealed that inhibition of S1PR3 reduced neutrophil infiltration in the lung and suppressed IL-6 and TNF-α production in vitro and in vivo compared to those in the LPS group. Accordingly, Murakami et al. [35] reported that the knockout of S1PR3 attenuates pulmonary inflammation in a bleomycin-induced lung injury mouse model. Eskan et al. [36] reported that LPS induces the production of IL-6 and IL-8 by activating S1PR3 in epithelial cells. These results indicated that S1PR3 is an important regulator of inflammatory responses.

Endothelial dysfunction is a hallmark of ARDS [6]. The endothelium is a monolayer of cells that controls the permeability of fluids, proteins and blood cells [37]. In ARDS, endothelial barrier permeability increases as the monolayer integrity is disrupted, allowing fluid to move into the interstitial tissue, further decreasing gas exchange and inducing hypoxia [2, 6]. Endothelial cell‒cell adhesions such as AJs, tight junctions (TJs) and gap junctions (GJs) regulate barrier permeability [38]. Among them, AJs have more significant microvascular effects, and the expression of vascular endothelial cadherin (VE-cadherin) at endothelial AJs is essential for endothelial barrier function [39]. Specifically, the structure of AJs in endothelial tissue involves interactions among VE-cadherin, β-catenin and the actin cytoskeleton [40]. In the present study, we found that in the LPS-induced ARDS mouse model and the LPS-stimulated cell model, the inhibition of S1PR3 significantly resotred the expression of VE-cadherin and β-catenin, as shown by both WB and IF. In addition, we used rhodamine phalloidin to stain the cytoskeleton, and found that the inhibition of S1PR3 significantly reduced the LPS-induced disruption of the cytoskeleton. Moreover, Yasuda et al. [41] reported that the S1PR3 mediated the VE-cadherin mRNA levels in two types of endothelial cells. In a murine ventilator-induced lung injury model, S1PR3 induced endothelial barrier loss through ADAM10-mediated VE-cadherin cleavage [42]. Although we demonstrated that the inhibition of S1PR3 protects endothelial AJs, the effects of TJs and GJs cannot be ignored, as these cell junctions may interact. Thus, further investigation of this issue is needed.

The NF-κB pathway is considered the central regulator of inflammation and endothelial function in various diseases [28], and our previous study revealed that the NF-κB pathway is important in ARDS [43]. NF-κB has five subunits, and the p65 protein is the key transcriptionally active component. IκBα is a crucial controller of NF‐κB p65 activation and is phosphorylated by IKKβ proteins to release active NF‐κB p65 into the nucleus, thereby promoting the transcription of proinflammatory cytokines and chemokines [44]. In endothelial cells, activated NF-κB contributes to VCAM-1 expression to mediate endothelial inflammation and damage [45]. Previous research has indicated an association between S1PR3 and the NF-κB pathway. Yan et al. [46] reported that the NF-κB pathway participates in S1PR3-mediated human renal cell carcinoma progression, and Dong et al. [47] reported that the S1PR3- NF‐κB axis may be a therapeutic target for ischaemic stroke. Moreover, previous studies have revealed the NF-κB-dependent proinflammatory role of the S1P/S1PR3 axis in lung adenocarcinoma cells, and the activation of S1PR3 facilitates tumour cell proliferation [48, 49]. Our results suggested that LPS increased the relative protein expression of p-NF‐κB p65/NF‐κB p65, p-IκBα/IκBα, p-IKKβ/IKKβ and VCAM-1/GAPDH in vitro and in vivo, while these changes were reversed by S1PR3 inhibition. Furthermore, the administration of an NF‐κB activator abolished the S1PR3 inhibition-mediated reductions in the inflammatory response and endothelial injury in the LPS-induced ARDS mouse model and the LPS-stimulated cell model. These results indicated that the NF‐κB pathway may be involved in the S1PR3-mediated protective effect against ARDS.

As the main powerhouses of cells, mitochondria are crucial for regulating the inflammatory response and barrier function in endothelial cells [50]. Mitochondrial oxidative phosphorylation is the main pathway for ATP production and is dependent on the functioning of the mitochondrial membrane potential and the activity of the respiratory chain complexes [51]. Consistent with previous studies [52], we found that LPS suppressed mitochondrial oxidative phosphorylation. S1PR3 inhibition decreased the ROS levels in lung tissues and the mitochondrial ROS levels in ECs, and increased the aggregate-to-monomer ratio. In addition, ATP production and the mitochondrial respiratory chain comple activity were restored after S1PR3 inhibition. The complex V inhibitor oligomycin abolished the protective effect of S1PR3 inhibition on ARDS. Therefore, mitochondrial oxidative phosphorylation may be involved in the protective effect mediated by S1PR3 in ARDS.

This study has several limitations. First, the study used only one cell line, HUVECs, and the results may not fully reflect how S1PR3 inhibition alleviates ARDS. Thus, future studies should include other endothelial cell lines, such as pulmonary microvascular endothelial cells, to further investigate the association between S1PR3 and ARDS. Second, the role of S1PR3 inhibition in protecting against inflammation and the endothelial cell barrier in ARDS should be further explored in conventional S1PR3 knockout mice. Third, these results may not be able to be directly applied to humans since the S1PR3 inhibition experiments were only conducted on mouse and in vitro cell cultures in our present study.

## Conclusions

In the present study, we found that the S1PR3 level was increased in mice with LPS-induced ARDS. We further demonstrated that S1PR3 inhibition alleviates the inflammatory response and repairs the EC barrier in ARDS through the NF-κB pathway and mitochondrial oxidative phosphorylation. Taken together, these results suggested that S1PR3 could be a potential therapeutic target for ARDS.

### Supplementary Information

**Additional file 1: Figure S1.** Transcriptomic profiling of lung tissues in mice with LPS or LPS + TY-52156. Mice were intratracheally injected with LPS (5 mg/kg), and TY-52156 (10 mg/kg) was injected intraperitoneally 1 h before LPS injection. Lung tissues were harvested at 48 h after LPS stimulation. A Heatmap of RNA-seq data. B Gene ontology enrichment analysis for all differentially expressed mRNAs. C KEGG signaling pathway enrichment analysis for all differentially expressed mRNAs. D GSEA analysis of the ‘Inflammatory response’ pathway between LPS group and LPS + TY-52156 group. E GSEA analysis of the ‘TNF-α signaling via NF-κB’ pathway between LPS group and LPS + TY-52156 group.

## Data Availability

Data supporting the conclusions of this article are included within the article. The datasets used and analyzed during the current study are available from the corresponding author on reasonable request.

## Abbreviations

AAV: Adenoviral-associated virus
ARDS: Acute respiratory distress syndrome
AJ: Adherens junctions
ATP: Adenosine triphosphatase
BCA: Bicinchoninic acid
DAPI: 4',6-Diamidino-2-phenylindole
DHE: Dihydroethidium
EBD: Evans blue dye
EC: Endothelial cell
ELISA: Enzyme-linked immunosorbent assay
ECL: Enhanced chemiluminescence
FITC: Fluorescein isothiocyanate
GJ: Gap junction
H&E: Hematoxylin and eosin
HUVEC: Human umbilical vein endothelial cell
IHC: Immunohistochemistry
IL: Interleukin
LPS: Lipopolysaccharide
MMP: Mitochondrial membrane potential
mtROS: Mitochondrial ROS
PBS: Phosphate buffer saline
ROC: Receiver operating characteristic curve
S1P: Sphingosine 1-phosphate
S1PR3: Sphingosine-1-phosphate receptor 3
siRNA: Small interfering RNA
TJ: Tight junction
TNF: Tumor necrosis factor
TUNEL: Terminal deoxynucleotide transferase-mediated dUTP nick end labeling
