# Supplementary figures and images for: S1PR3 inhibition protects against LPS-induced ARDS by inhibiting NF-κB and improving mitochondrial oxidative phosphorylation
Source: J Transl Med. 2024 Jun 5;22:535. doi: 10.1186/s12967-024-05220-9 (PMC11151509; doi:10.1186/s12967-024-05220-9)

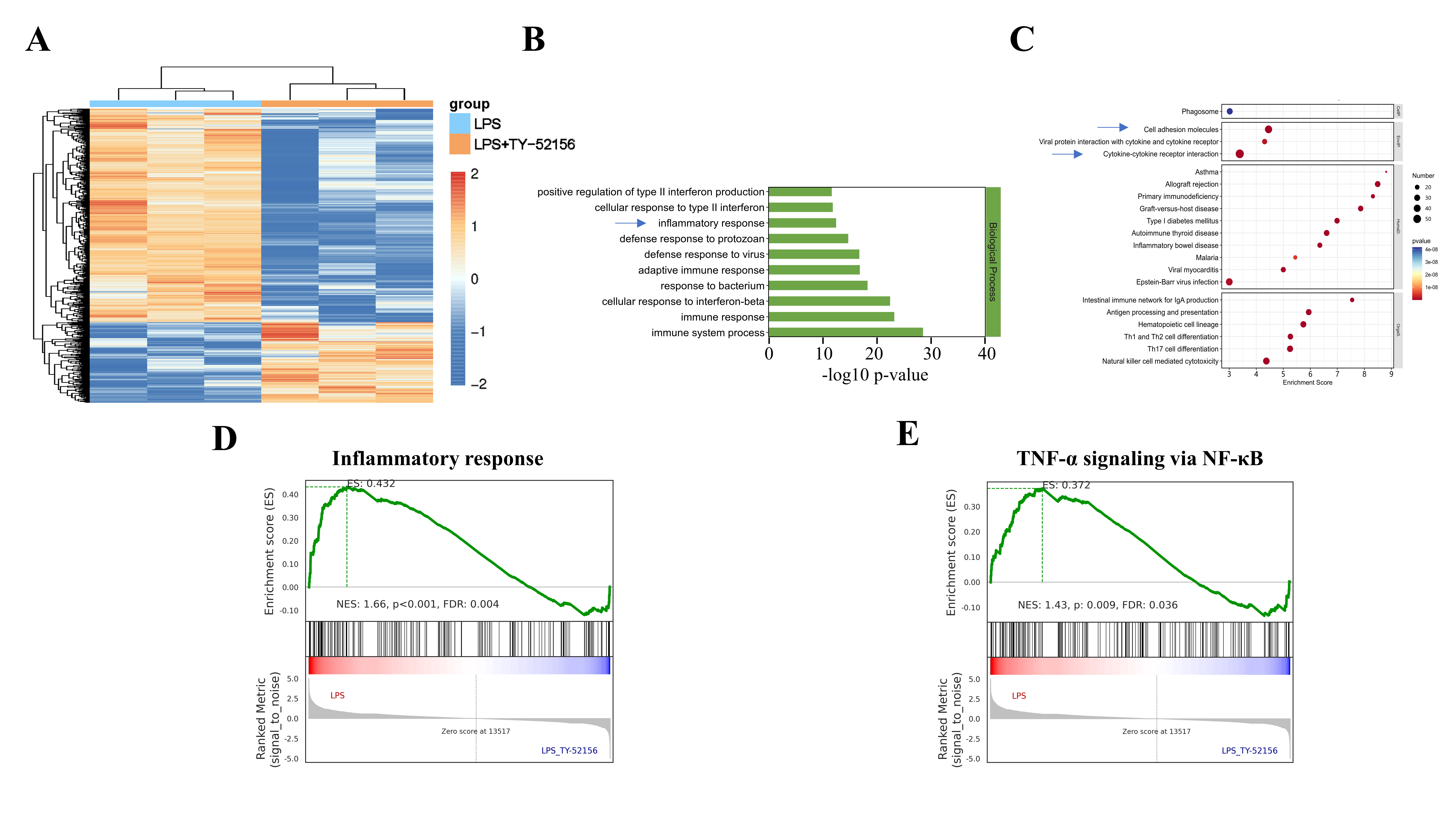

Supplement: Supplementary file 1 — Additional file 1: Figure S1. Transcriptomic profiling of lung tissues in mice with LPS or LPS + TY-52156. Mice were intratracheally injected with LPS (5 mg/kg), and TY-52156 (10 mg/kg) was injected intraperitoneally 1 h before LPS injection. Lung tissues were harvested at 48 h after LPS stimulation. A Heatmap of RNA-seq data. B Gene ontology enrichment analysis for all differentially expressed mRNAs. C KEGG signaling pathway enrichment analysis for all differentially expressed mRNAs. D GSEA analysis of the ‘Inflammatory response’ pathway between LPS group and LPS + TY-52156 group. E GSEA analysis of the ‘TNF-α signaling via NF-κB’ pathway between LPS group and LPS + TY-52156 group. [file 12967_2024_5220_MOESM1_ESM.tif]
